# DCDB 2.0: a major update of the drug combination database

**DOI:** 10.1093/database/bau124

**Published:** 2014-12-23

**Authors:** Yanbin Liu, Qiang Wei, Guisheng Yu, Wanxia Gai, Yongquan Li, Xin Chen

**Affiliations:** ^1^Department of Bioinformatics, College of Life Sciences and ^2^Institute of Biochemistry, College of Life Sciences, Zhejiang University, Hangzhou 310058, P.R. China

## Abstract

Experience in clinical practice and research in systems pharmacology suggested the limitations of the current one-drug-one-target paradigm in new drug discovery. Single-target drugs may not always produce desired physiological effects on the entire biological system, even if they have successfully regulated the activities of their designated targets. On the other hand, multicomponent therapy, in which two or more agents simultaneously interact with multiple targets, has attracted growing attention. Many drug combinations consisting of multiple agents have already entered clinical practice, especially in treating complex and refractory diseases. Drug combination database (DCDB), launched in 2010, is the first available database that collects and organizes information on drug combinations, with an aim to facilitate systems-oriented new drug discovery. Here, we report the second major release of DCDB (Version 2.0), which includes 866 new drug combinations (1363 in total), consisting of 904 distinctive components. These drug combinations are curated from ∼140 000 clinical studies and the food and drug administration (FDA) electronic orange book. In this update, DCDB collects 237 unsuccessful drug combinations, which may provide a contrast for systematic discovery of the patterns in successful drug combinations.

**Database URL:** http://www.cls.zju.edu.cn/dcdb/

## Introduction

Ever since the existence of ‘chemoreceptor’ was postulated by Paul Ehrlich in the 1870s, the search for new medicines was focused on the identification of bioactive compounds that can selectively act on molecular targets important in disease etiology. To this date, ∼1700 drugs have been found to interact with more than 4000 proteins (1). These medicines have greatly improved our quality of life. However, accumulating evidence has suggested that this one-drug-one-target model is limited in many aspects (2). First, small compounds typically have imperfect selectivity. They can interact with various ‘off-targets’ besides their designated targets, which may cause unexpected side effects. Second, drug targets often have important normal physiological functions in addition to their roles in disease etiology. Unwarranted interference of a target’s normal physiological function may also create side effects. Last but not the least, in many diseases, hardly any single target can be found to produce sufficient therapeutics. These difficulties lead to a bottleneck for us to achieve novel or better therapeutics with the one-drug-one-target paradigm.

To overcome these difficulties, a viable approach is to use combinations of drugs that work synergistically to potentiate therapeutic effects, and/or, work antagonistically to alleviate side effects. In this context, a drug combination is a recipe of two or more active ingredients, with a fixed dose relationship, used for treating one specific condition/indication. Drug combinations have already showed advantage in treating complex and refractory diseases, e.g. acquired immune deficiency syndrome (AIDS) (3) and cancer (4). In fact, therapies based on coordinated actions of multiple compounds have been used as early as the dawn of medicine. Traditional medicines, made of natural products containing many distinctive compounds, are still widely used today. It has been demonstrated that some of these mixtures are capable of providing unique clinical benefits that cannot be achieved by any of our modern medical approaches (5). In these cases, synergies between compounds were shown to be critical.

An important step to develop our current single-target drug discovery paradigm into that of designing multicomponent therapeutics is to understand the science behind beneficial drug interactions. To facilitate research in this direction, the drug combination database (DCDB) was first launched in 2010, which collected and organized information on 497 known examples of drug combinations. With rapid advances in drug combination research (6–9), a large amount of data from clinical trials and drug regulatory agencies become available. Here, we present the second major release of DCDB, which includes 866 new drug combinations (1363 in total), consisting of 904 distinctive components. Among these drug combinations, 682 were curated from clinical trials with published reports, 152 were curated from the FDA orange book and 32 were curated from PubMed articles. In this update, DCDB also includes 237 unsuccessful drug combinations, which may provide a contrast for systematic discovery of the patterns in successful drug combinations. All DCDB data and documentation can be accessed at http://www.cls.zju.edu.cn/dcdb/ (the trailing slash is needed).

## Data curation

### Data sources

Drug combinations and their usages were collected from the ClinicalTrials.gov database, FDA orange book and PubMed database. ClinicalTrials.gov is a registry and result database for clinical studies conducted in 185 countries. The records in ClinicalTrials.gov are mostly interventional studies, with a small proportion of them being observational programs. Each record in ClinicalTrials.gov shows the summary information of a study, including indication/condition, intervention type (medical product, behavior or procedure), description, design, location, sponsor, etc. The FDA orange book provides information on approved drug combinations, including their ingredients, application, etc.

The chemical, pharmaceutical and biological information about the components in drug combinations were curated from four databases, DrugBank (1), kyoto encyclopedia of genes and genomes (KEGG) (10), PubChem (11) and therapeutic target database (TTD) (12). DrugBank is a data source providing chemical information for 6825 drugs, which include 1541 FDA-approved small molecules, 150 FDA-approved biotech products, 86 nutraceuticals and 5082 experimental drugs. DrugBank also provides information about 4323 non-redundant drug-target proteins. PubChem supplies chemical information for almost all available small compounds. KEGG provides metabolic information for drugs and protein annotation for drug targets. TTD is a database specialized in annotation of known and explored macromolecular drug targets. Annotation of drug-target proteins was also retrieved from Uniprot database (13). In cases that information about a drug component was unavailable in the earlier databases, this drug component would be annotated based on related research articles in PubMed.

### Drug combination curation

From ClinicalTrials.gov, 149 273 records of clinical trials were retrieved. To collect drug combination studies, therapeutic agents involved in the trialed arms were mapped to a standard nomenclature using the MetaMap tool (14). MetaMap recognizes unified medical language system (UMLS) (15) concepts in text, through a knowledge-intensive approach based on symbolic computing, natural language processing and linguistic techniques (14). All drug names were mapped to UMLS concept IDs with semantic types ‘biologically active substance’, ‘pharmacologic substance’, ‘antibiotic’, ‘clinical drug’ and ‘steroid’, where a mapping score cut-off of 850 was used. All drug name mappings were manually verified, and ∼5% of the mappings were corrected according to the original registry information. In this manual validation process, studies involving only vaccines and vitamins were discarded. In addition, drug combinations involving more than seven components were ignored, because their mutual interactions were likely too complex for this initial stage of drug combination study. As a result, we collected 8245 records describing interventional trials, which had more than one distinctive agent in at least one arm. Then, ongoing and terminated trials were removed based on their status and/or trial reports. Finally, 783 trials were found with known clinical results, which included 682 new drug combinations. The electronic version of the FDA orange book was downloaded and curated for approved drug combinations, from which 152 approved combinations were collected. Furthermore, 32 new drug combinations were curated from PubMed literature.

Together with existing data in DCDB, this update presents 1363 drug combinations from 1209 clinical trials, 200 preclinical studies and 404 FDA-approved products. These drug combinations have 1813 distinct usages, which were classified as ‘efficacious’, ‘need further study’ or ‘non-efficacious’ according to their FDA-regulatory status, clinical trial outcomes and/or related literature. The individual drug components in DCDB include 806 small compounds and 98 biotech drugs, which interact with 814 drug targets.

### Classification of drug interaction in drug combinations

Similar to the previous version of DCDB, drug interactions in drug combinations were categorized with a two level system. On the first level, drug interactions are divided into pharmacodynamic interactions and pharmacokinetic interactions. Pharmacodynamic interactions are those in which the effects of one drug are changed by the presence of another, at its site of action; pharmacokinetic interactions are those in which the processes of absorption, distribution, metabolism and excretion of one drug are altered by the other. On the second level, pharmacodynamic interactions are further grouped into four classes based on their ‘sites of action’, which are ‘acting on the same target’, ‘acting on different targets in the same biological process’, ‘acting on different targets in related processes’ and ‘acting on different targets in processes of yet unknown relations’. Similarly, pharmacokinetic interactions are further grouped into four classes, which are ‘regulation of drug transport or permeation’, ‘regulation of drug distribution or localization’, ‘drug metabolism interaction’ and ‘drug elimination interaction’. It shall be noted that the earlier categories of drug interaction are not mutually exclusive. A drug combination may have multiple pharmacodynamic and pharmacokinetic interactions at the same time.

### Web interface and implementation

The web interface of DCDB supports query by individual drug, drug combination, usage and drug target. All text queries support the use of wild character ‘*’ (representing a string of any length) and ‘?’ (representing a single character). For each drug combination, DCDB shows its therapeutic components, combined activity/trialed indication, potential mechanisms of drug interaction, drug interaction classifications and regulatory status, together with literature reference. For each small compound, DCDB shows its chemical and pharmacological information, with cross-links to DrugBank, PubChem, KEGG, RxList and wiki. For each drug target, DCDB shows its protein annotations, pathway affiliations in Reactome (16) and Biocarta (17), as well as cross-links to protein interaction databases IntAct (18) and human interactome resource (HIR) (19).

The DCDB database is implemented with oracle 11.2 and Glassfish web server 2.1. Users intended to conduct computational analysis of DCDB can download its oracle dump and plain txt data files. The database schema and related documents are provided in the website. The DCDB website also provides a number of data summary spreadsheets.

## Database overview

In DCDB, there are drug combinations in different development stages. As shown in Table 1, 22% of the drug combinations in DCDB are FDA approved. Thirty-two percentage of the drug combinations are in early development stages, i.e. preclinical, Phase I and Phase II. Thirty-eight percentage of the drug combinations are in late development stages, i.e. Phase III and Phase IV. Among the 1813 drug combination usages, 1445 were reported to reach their study criteria in clinical trials and were annotated as ‘efficacious’. Two hundred and thirty-seven usages failed to reach their study criteria and were annotated as ‘non-efficacious’. The non-efficacious rate for drug combination usages in early development stages is 15.1%, and, that in late development stages is similar, i.e. 17.8%. In addition, there are 131 drug combination usages, for which clinical trials reported ambiguous conclusions or produced statistically insignificant results. These usages were annotated as ‘need further study’.

**Table 1.** Composition of drug combination usages in DCDB

Development stage	Number of drug combination usages	Efficacious	Need further study	Non-efficacious
Approved	404	404	0	0
Preclinical	198	169	10	19
Phase 0	1	1	0	0
Phase I	42	23	16	3
Phase I&II	42	31	6	5
Phase II	261	189	22	50
Phase II&III	44	19	13	12
Phase III	460	353	26	81
Phase IV	238	170	24	44
No phase information	123	86	14	23
Total	1813	1445	131	237

No phase information: the phase information of the clinical trial is not specified or not available. Efficacious: the overall clinical outcome of a drug combination shows significant improvement in efficacy or safety. Need further study: the overall clinical outcome of a drug combination shows no significant improvement or the improvement is not consistent between groups. Non-efficacious: the overall clinical outcome of a drug combination shows no improvement if compared with individual drugs/current therapies or it leads to unacceptable side effects/toxicity.

The conditions/indications of drug combination usages in DCDB are mapped to the disease classification system ICD10CM. About 61% of all drug combination usages belong to four therapeutic areas, which are ‘neoplasms’ (∼26%), ‘respiratory system, endocrine, nutritional and metabolic disease’ (∼14%), ‘diseases of the blood and blood forming organs, circulatory system’ (∼12%) and ‘infections and parasitic diseases’ (∼9%).

## Successful drug combinations

Drug combinations have been extensively used for clinical applications and showed advantage over using individual drugs. Combination of distinct drugs can help to improve therapeutic efficacy by overcoming the redundancy and robustness of pathogenic process and/or lowering the risk of side effects by decreasing the dosages of individual drugs at an equal or increased level of efficacy. The exact mechanisms underlying beneficial drug interactions are often unclear but it can broadly be grouped into pharmacokinetic and pharmacodynamic interactions (20).

A well-known example of pharmacokinetic interaction is the combination of ticarcillin and clavulanate potassium. Ticarcillin is an antibiotic of the penicillin-family, and clavulanate potassium is a form of clavulanic acid, which inhibits beta-lactamase and prevents ticarcillin metabolism (21). Another example is the combination of probenecid and ciprofloxacin. Probenecid modulates the transport of ciprofloxacin at the proximal and distal renal tubule, prolonging the serum retaining time of ciprofloxacin (22).

Pharmacodynamic interactions may result in synergistically increased efficacy, reduced resistance and reduced side effect. For example, the combination of celecoxib and emodin exhibited synergistic growth repression on cholangiocarcinoma cells. Celecoxib inhibits cancer growth by inactivating protein kinase B (also known as AKT), which suppresses apoptosis. Emodin inhibits cancer growth by suppressing tyrosine kinases and down regulating AKT. Emodin complements the inactivation of AKT by celecoxib and enhances the induction of apoptosis (23). In the combination of erlotinib and cetuximab, erlotinib is an epidermal growth factor receptor (EGFR) inhibitor, typically used in non-small-cell lung cancer. However, the acquired T790M mutation of EGFR leads to erlotinib resistance. Cetuximab is a chimeric monoclonal antibody to EGFR, supplementing cetuximab within erlotinib therapy was found to overcome the acquired resistance (24). In the combination of rosiglitazone and exenatide, rosiglitazone is an efficacious anti-diabetic drug but it increases the risk of myocardial infarction. Exenatide was found to reduce rosiglitazone-associated myocardial infarction when used in combination (25).

An interesting consequence of pharmacodynamic interaction is that using a drug in combination may expand its known therapeutic area. For example, an anti-psychotic drug chlorpromazine and an anti-protozoal drug pentamidine, neither of which showed any anti-tumor activity when tested alone, together prevented tumor growth more effectively than paclitaxel (26). Metformin is currently a first-line drug for type 2 diabetes, but a growing amount of evidence suggested that metformin might be associated with decreased risk of cancer, and with a better response to cancer chemotherapy (27, 28). Simvastatin is a hypolipidemic drug used to control elevated cholesterol level, but simvastatin was also found to improve chemotherapy outcome in metastatic colorectal patients (29). Furthermore, research in the past two decades revealed that continued oxidative stress leads to chronic inflammation, which predisposes many chronic diseases, including cancer, diabetes, cardiovascular disease, neurological disease and pulmonary disease (30). This knowledge also highlights a great number of possibilities to combine drugs from different therapeutic classes for systematic management of chronic diseases (31).

## Unsuccessful drug combinations

Not all drug combinations produced expected outcomes. Sometimes they produce no improvement of therapeutics over individual drugs and/or unacceptable side effects. For example, dasatinib is an inhibitor of Src family kinases that functions in prostate cancer progression. Inhibiting Src family kinases is known to suppress cancer cell proliferation, invasion and migration (32). Another drug, docetaxel, stabilizes tubulin, which prevents microtubule disassembly and leads to cell-cycle arrest and apoptosis. The therapeutic mechanisms of these two drugs seemed complementary. However, adding dasatinib into standard docetaxel therapy did not improve survival and other major clinical endpoints in a Phase III trial (33). Similarly, temsirolimus and interferon-alpha are both individually used for advanced renal-cell carcinoma. However, when used in combination, the overall survival is not improved if compared with using interferon alone and is even inferior if compared with using temsirolimus alone (34). On the other hand, drug combinations may also lead to unacceptable side effects. For example, the combination of bevacizumab and temsirolimus showed much higher toxicity than anticipated, and therefore was not recommended for first-line treatment of patients with metastatic renal-cell carcinoma (35). In another example, using sorafenib and pegylated interferon α-2 b together to treat metastatic melanoma produced more cutaneous side effects than using individual agents (36).

In DCDB, ∼20% of the drug combination usages are approved, whereas 13% of the drug combination usages are reported to be non-efficacious. These data indicate that not all seemingly reasonable drug combinations would produce better efficacy or safety. Although examples of successful drug combinations may help us summarize the patterns in beneficial drug combinations, non-efficacious drug combinations, especially Phase III/IV failures, may help us understand the types of drug interactions that should be avoided. DCDB provides both types of examples in different development stages.

## Research in drug combinations

Using drug combination is a promising strategy to combat complex disorders. To this date, clinical developments of drug combinations are typically through trial and error or guided by insight into the dysregulated signaling pathways in specific diseases. In this direction, computational prediction and high-throughput screening of potentially beneficial drug combinations made notable progresses (9, 26, 37–39). For example, Miller *et al.* (40) performed a combinatorial drug screen in a dedifferentiated liposarcoma (DDLS)-derived cell line, and identified cyclin-dependent kinase 4 (CDK4) and insulin-like growth factor 1 receptor (IGF1R) as synergistic drug targets. Combined inhibition of CDK4 and IGF1R was confirmed to cooperatively suppress the activation of AKT pathway and consequently suppress DDLS. In this example, network models constructed from context-specific proteomic measurements of systematically perturbed cancer cells revealed cancer-specific signaling mechanisms and informed the design of this effective combination. In addition, Lee *et al.* (9) presented a genomics and bioinformatics system called the combination drug assembler, which predicts effective drug combinations based on individual drug-induced transcriptional profiles. Their predictions were successfully validated in non-small-cell lung cancers cells and triple-negative breast cancer cells.

Novel drug interaction quantification methods are also being actively developed for drug combination research (41, 42). Pritchard *et al.* (41) integrated an RNAi-based experimental system with complementary informatics tools, which were used to measure drug interactions. In addition, safety assessment remains an independent challenge in drug combination research. A systematic protein-protein interaction network analysis approach was presented to predict dangerous pharmacodynamic drug interactions (43). When evaluated with known adverse events data, this approach showed an accuracy of 82% and a recall of 62%.

## Concluding remarks

Using drug combinations is a promising strategy to combat complex diseases, which opens new possibilities to improve efficacy and reduce side effects, at a systems level. However, mechanisms and principles that underlie combined therapeutic benefits are still elusive. In practice, drug combinations did not always produce intended improvements. They often produced no therapeutic improvement and/or unexpected severe side effects. To elucidate the principles behind successful drug combinations, examples of known successful and unsuccessful drug combinations are needed. DCDB V2.0 presents 1363 cases of drug combinations to facilitate this line of research.

## Funding

National Basic Research Program of China (grant number 2012CB944900). National Natural Science Foundation of China (NSFC) (grant number 31071161). China Postdoctoral Science Foundation (grant number 2012M521164). Funding for open access charge: National Basic Research Program of China (2012CB944900).

*Conflict of interest.* None declared.
